# Plasma and Red Cell Reference Intervals of 5-Methyltetrahydrofolate of Healthy Adults in Whom Biochemical Functional Deficiencies of Folate and Vitamin B**_12_** Had Been Excluded

**DOI:** 10.1155/2014/465623

**Published:** 2014-01-15

**Authors:** Agata Sobczyńska-Malefora, Dominic J. Harrington, Kieran Voong, Martin J. Shearer

**Affiliations:** The Nutristasis Unit, The Centre for Haemostasis and Thrombosis, GSTS Pathology (Part of King's Healthcare Partners), St. Thomas' Hospital, London SE1 7EH, UK

## Abstract

5-Methyltetrahydrofolate (5-MTHF) is the predominant form of folate and a strong determinant of homocysteine concentrations. There is evidence that suboptimal 5-MTHF availability is a risk factor for cardiovascular disease independent of homocysteine. The analysis of folates remains challenging and is almost exclusively limited to the reporting of “total” folate rather than individual molecular forms. The purpose of this study was to establish the reference intervals of 5-MTHF in plasma and red cells of healthy adults who had been prescreened to exclude biochemical evidence of functional deficiency of folate and/or vitamin B_12_. Functional folate and vitamin B_12_ status was assessed by respective plasma measurements of homocysteine and methylmalonic acid in 144 healthy volunteers, aged 19–64 years. After the exclusion of 10 individuals, values for 134 subjects were used to establish the upper reference limits for homocysteine (13 **μ**mol/L females and 15 **μ**mol/L males) and methylmalonic acid (430 nmol/L). Subjects with values below these cutoffs were designated as folate and vitamin B_12_ replete and their plasma and red cell 5-MTHF reference intervals determined, *N* = 126: 6.6–39.9 nmol/L and 223–1041 nmol/L, respectively. The application of these intervals will assist in the evaluation of folate status and facilitate studies to evaluate the relationship of 5-MTHF to disease.

## 1. Introduction

**Figure 1:**
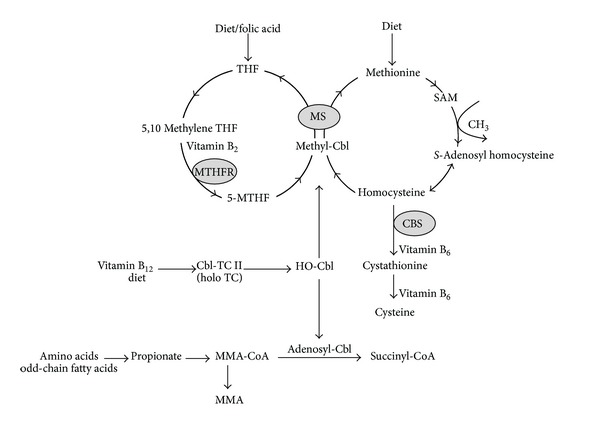
Homocysteine, folate, and vitamin B_12_ metabolism. THF (tetrahydrofolate), 5-MTHF (5-methyltetrahydrofolate), MTHFR (methylene tetrahydrofolate reductase), MS (methionine synthase), CBS (cystathionine beta-synthase), SAM (*S*-adenosyl methionine), Cbl (cobalamin), TC II (transcobalamin), holo TC (holotrascobalamin), OH-Cbl (hydroxocobalamin), MMA-CoA (methylmalonyl-CoA), and MMA (methylmalonic acid).

5-MTHF, the predominant form of folate (vitamin B_9_) in plasma and red cells, is a substrate for the methionine synthase and vitamin B_12_ (methylcobalamin form—methyl-Cbl) mediated conversion of homocysteine (tHcy) to methionine (Figure 1). Suboptimal 5-MTHF availability leads to an increase in circulating homocysteine (hyperhomocysteinaemia) which has been associated with many diseases and health complications including cardiovascular disease [1, 2]. There is also evidence to suggest that 5-MTHF deficiency may be a cardiovascular risk factor independent of homocysteine [3, 4].

In the plasma of healthy humans, 5-MTHF typically constitutes 80–90% of total folate [5, 6]. Circulatory concentrations of 5-MTHF are partly dependant on methylenetetrahydrofolate reductase (MTHFR) genotype [7, 8]. Conversely, the 5-MTHF content of red cells has been reported to vary greatly [9, 10].

Tissue folate status is typically assessed by measurement of “total” folate concentration in blood because commonly available assays are unable to differentiate between the various circulatory forms. Analysis of folate in red cells is considered to be a strong indicator of folate adequacy because it reflects intracellular status and is not influenced by recent or transient changes in dietary folate intake. Traditionally a value of 317 nmol/L (140 *μ*g/L) for red cell folate (RCF) has been considered as the cutoff concentration for folate adequacy [11]. Serum levels <7.9 nmol/L (<3 *μ*g/L) usually indicate folate insufficiency. However, there is little consensus for the reference intervals for plasma and red cell folate [12]. Furthermore, methodology bias is very common in folate assays and particularly pronounced for RCF values, as a consequence of variation in approach to the preparation and storage of red cell lysates [12]. This lack of harmonisation of folate methods prevents the interlaboratory adoption of reference intervals.

The purpose of this study was to establish reference intervals for plasma and red cell 5-MTHF in adults, using a fully validated HPLC assay, after excluding subjects in whom functional folate and/or vitamin B_12_ deficiency was biochemically indicated by elevated concentrations of tHcy and/or MMA, respectively. The basis of an elevated MMA concentration as a sensitive functional indicator of vitamin B_12_ deficiency is that 5-deoxyadenosylcobalamin is an essential cofactor for the conversion of methylmalonyl-CoA to succinyl-CoA. In vitamin B_12_ insufficiency/deficiency (5-deoxyadenosylcobalamin form) the excess of methylmalonyl-CoA is hydrolysed to MMA causing the circulatory concentration of MMA to increase (Figure 1) [13].

## 2. Materials and Methods

### 2.1. Study Participants and Study Design

One hundred and forty-four volunteers, aged 19–64 years, were recruited from members of staff from St. Thomas' Hospital by advertisement. Subjects were excluded from participation if they were pregnant or taking vitamin supplements or drugs (e.g., phenytoin) known to interfere with folate or homocysteine metabolism. Recruited subjects were then screened to assess their functional folate and vitamin B_12_ status. This led to the exclusion of ten subjects who had either outlying values of tHcy and/or MMA or who admitted to previously undeclared use of relevant dietary supplements and/or drugs. Of the remaining 134 subjects, those with tHcy and MMA values above their respective 97.5th percentiles were deemed to be potentially folate or vitamin B_12_ deficient and were excluded from our reported reference intervals for 5-MTHF. The study was approved by St. Thomas' Hospital Local Research Ethics Committee and written consent was obtained from all participants.

### 2.2. Blood Collection and Analytical Methods

Nonfasting, venous blood samples were collected into EDTA-containing tubes and immediately placed on ice and protected from light. Following hematocrit determinations, lysates were prepared by the addition of 100 *μ*L whole blood to 900 *μ*L of 5 g/L ascorbic acid solution. Plasma was prepared by centrifugation. All samples were stored at –70°C until analysis.

Plasma tHcy was measured by automated fluorescence polarization immunoassay (IMx, Abbott Laboratories). The intra- and inter-CVs for this assay were 2.0% and 2.7%, respectively. Plasma MMA was analysed using HPLC [14]. The differences of duplicate analysis of 16 samples ranged from 0.0 to 10.9%, while the interassay CVs for five samples (concentration range: 177–1114 nmol/L) were between 6 and 12%.

Plasma and red blood cell 5-MTHF were measured by HPLC as previously described [15]. In brief, 4-aminoacetophenone was used as an internal standard [16] and Bond Elut C_18_ (100 mg, 1 mL reservoir) cartridges (Varian Inc.) were utilised for SPE with an elution strategy based on that of Pfeiffer et al. [6] with some in-house modifications. Sample components were separated using a ACE C_18_, 3 *μ*m column (125 × 4.6 mm) supplied by Hichrom, UK, with a mobile phase composition of 0.033 mol/L potassium phosphate buffer (pH 2.3) : acetonitrile : methanol (89 : 6.6 : 4.4, by volume) at a flow rate of 0.34 mL/min. The fluorescence detector wavelength settings were: excitation 290 nm and emission 365 nm.

A primary stock solution of 5-MTHF was prepared by dissolving ~5 mg of 5-MTHF powder in 10 mL of 20 mM potassium phosphate buffer pH 7.2 with 1 g/L cysteine. An aliquot (1 mL) of this stock solution was removed to determine the concentration by UV spectrophotometry and 90 mg of ascorbic acid powder was immediately added to the remaining stock solution. The absorbance of 5-MTHF was measured at 290 nm using the molar absorptivity value of 32000 L mol^−1 ^cm^−1^ after 1 in 40 and 1 in 33.3 dilution with 20 mmol/L potassium phosphate buffer pH 7.2 containing 1 g/L cysteine.

The primary stock solution (1018.70 *μ*mol/L) was diluted 1 in 5 in 10 g/L ascorbic acid and this stock solution was aliquoted and stored at −70°C (secondary stock: 203.74 *μ*mol/L). One aliquot of the secondary stock solution was diluted further with 1 g/L ascorbic acid to the concentration of 20.37 *μ*mol/L (tertiary stock). This stock was used on the day of analysis to prepare a calibration curve of a minimum of four points.

The accuracy of our 5-MTHF calibration standard was checked against the Standard Reference Material (SRM) 1955 (National Institute of Standards & Technology, USA) which included three reference samples with certified 5-MTHF concentrations (uncertainties) of 4.26 ± 0.25, 9.73 ± 0.24, and 37.1 ± 1.4 nmol/L [17]. These reference samples were used to construct a calibration curve to verify the 5-MTHF concentration of our secondary stock solution with the theoretical concentration (by UV spectroscopy) of 203.74 *μ*mol/L. The calculated concentration of our secondary stock using the SRM 1955 generated standard curve was 196.13 *μ*mol/L (3.6% difference). In another analytical run the standard curve was prepared by serial dilutions of the 20.37 *μ*mol/L 5-MTHF tertiary stock (as in the typical HPLC run) and SRM 1955 samples were analysed. The CVs for the means derived from the values assigned by the manufacturer of SRM 1955 and obtained in these analyses ranged from 2.1 to 5.9% for samples with the concentration of 4.26 and 37.1 nmol/L, respectively.

The 5-MTHF red cell folate concentration was calculated according to the formula

(1) 5-MTHF
  in  red  cells ={whole  blood  5-MTHF
   −[plasma  5-MTHF
(1−hematocrit)]}  ×hematocrit−1

[4, 18].

### 2.3. Statistical Analysis

Normality of data was checked by the Kolmogorov-Smirnov test. Where the variables were not normally distributed; nonparametric tests or log_10_ transformed values were used. Subjects with outlying tHcy, MMA, and 5-MTHF values were excluded using the Tukey test [19, 20]. For the lower (where appropriate) and upper reference limits the 2.5th and 97.5th percentiles were used. The *z*-test was used to establish whether to partition reference values by sex [19, 21]. The independent samples Student's *t*-test was applied to compare the values between sexes. Confidence intervals for the lower and upper reference limits were obtained using the rank numbers [19, 22]. Pearson's or Spearman's correlations were carried out to examine relationships between two continuous variables. Results were considered statistically significant if the observed, two-sided *P* value was <0.05. Statistical analyses were carried out using SPSS for Windows (SPSS Inc., USA).

## 3. Results

### 3.1. Screening for Subjects with Outlying tHcy and MMA Results and the 97.5th Percentiles for tHcy and MMA

Of the 144 subjects initially recruited, 72 (50%) were represented by laboratory staff, 36 (25%) held administrative positions, 24 (16%) were from supporting services, and 12 (8%) held clinical posts. The majority of participants were Caucasian *N* = 107 (74%), with 20 (14%) of Asian and 17 (12%) of Afro-Caribbean origins. Ten of these subjects were excluded before the determination of the upper cutoff values for plasma tHcy and MMA. Three of these 10 subjects were taking vitamin supplements and one subject was taking opiates which are known to affect folate metabolism [23, 24]. Two subjects with tHcy >15 *μ*mol/L, on further investigation, were found to be taking medication that interfered with tHcy metabolism [23, 25]. The remaining four subjects were identified as outliers (Tukey method). Two of these four subjects had an MMA >1000 nmol/L (suggestive of vitamin B_12_ deficiency); one had a family history of pernicious anaemia whilst the other individual was on medications and consumed a vegetarian diet. One of these four subjects had a plasma tHcy of 25.7 *μ*mol/L with low MMA (121 nmol/L) suggesting folate deficiency and in another both markers were elevated. To the best of our knowledge no other volunteers participating in the study were taking vitamins or any drugs/medications interfering with folate/homocysteine metabolism. The remaining 134 subjects were used to establish the upper cutoff values for tHcy.

Plasma tHcy was normally distributed (*P* = 0.348), whilst plasma MMA was positively skewed (*P* = 0.007). There was a gender difference in tHcy concentrations (*P* = 0.01) with males having higher values. Plasma MMA concentration did not differ between genders (*P* = 0.440). After the exclusion of the upper 2.5th percentile, upper reference limits for tHcy were established as 13 *μ*mol/L (females, *N* = 72) and 15 *μ*mol/L (males, *N* = 62) and for MMA as 430 nmol/L (all 134 subjects).

### 3.2. Establishing Reference Intervals for 5-MTHF

**Figure 2:**
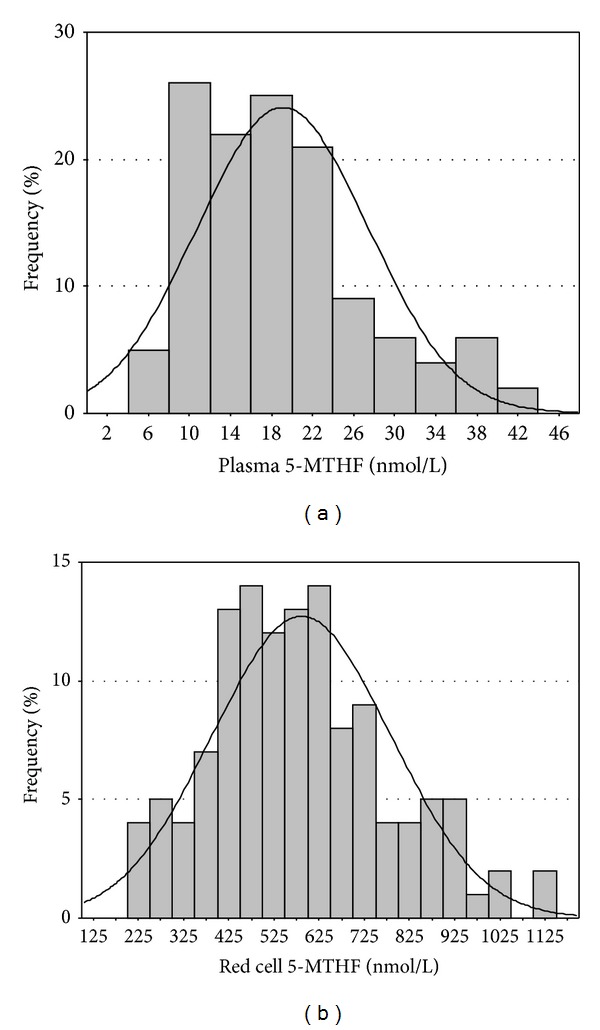
Distribution of the 5-MTHF concentrations in plasma and red cells.

Five subjects with functional deficiencies of folate and/or vitamin B_12_ as defined by elevated tHcy (>13 *μ*mol/L for females and >15 *μ*mol/L for males) and MMA (>430 nmol/L for both genders) were excluded from further analysis. In addition, three subjects had highly elevated red cells 5-MTHF of >1143 nmol/L (identified by the Tukey test as outlying values) and these subjects were also excluded. There were no outlying plasma 5-MTHF values. Hence, 126 subjects were eligible for the construction of the reference intervals for 5-MTHF. The ethnic distribution of these 126 subjects was 93 Caucasian (73%), 17 Afro-Caribbean (14%), and 16 Asian (13%). The proportion of women within these ethnic groups was 41% Caucasian, 65% Afro-Caribbean, and 44% Asian. The age and summary of all results for females and males used to derive 5-MTHF reference intervals is given in Table 1. Age and all the parameters measured followed a normal distribution. The values remained normally distributed after partition by sex. Reference intervals together with 90% confidence intervals for the lower and upper 95% reference limits are displayed in Table 2. The distribution of 5-MTHF concentrations in plasma and red blood cells is shown in Figure 2. Plasma 5-MTHF correlated with whole blood 5-MTHF (*r* = 0.565, *P* < 0.001), red cell 5-MTHF (*r* = 0.523, *P* < 0.001) but did not correlate with age (*r* = 0.087, *P* = 0.332), tHcy (*r* = −0.122, *P* = 0.172), or MMA (*r* = −0.103, *P* = 0.256). Red cell 5-MTHF correlated with whole blood 5-MTHF (*r* = 0.998, *P* < 0.001) and age (*r* = 0.204, *P* = 0.022) but did not correlate with tHcy (*r* = −0.172, *P* = 0.054) and MMA (*r* = −0.047, *P* = 0.605). Whole blood 5-MTHF, in addition to its correlations with age, plasma, and red cell 5-MTHF, correlated with tHcy (*r* = −0.181, *P* = 0.043).

## 4. Discussion

Reference intervals of 5-MTHF were determined for plasma (6.6–39.9 nmol/L) and red cells (223–1040 nmol/L) for healthy adults aged 19–64 years. Separate reference intervals are also shown for the whole blood 5-MTHF which can be utilised if plasma 5-MTHF is not available to correct for red cell 5-MTHF contents. Many laboratories performing the standard RCF assay do not adjust their results for folate contents in plasma. Although this approach reduces assay costs, it may provide misleading RCF status, especially when plasma folate concentrations are >45 nmol/L [26].

Subjects with likely functional folate and/or vitamin B_12_ deficiency/insufficiency were excluded from the construction of 5-MTHF reference intervals after the determination of cohort specific upper limits for plasma tHcy and MMA. The upper limits for plasma tHcy and MMA were established prior to removal of six subjects who on subsequent review were discovered to be taking previously nondeclared medications or supplements and four subjects with outlying tHcy and/or MMA values. This process is in agreement with expert recommendations [27]. The value for the upper reference limit for tHcy of 13 *μ*mol/L for females and 15 *μ*mol/L for males established in our current study is similar to the upper limits of 12.8 and 14.7 *μ*mol/L observed for females and males, respectively (nonsmokers aged 40–42 years with high folate intakes and low-moderate coffee consumption), resident in western Norway [28]. The difference of 2 *μ*mol/L between the upper tHcy reference limit for females and males is consistent with a study reported by Jacques et al. [29]. Although it has been well documented that tHcy is influenced by gender and age [29], laboratories often still choose to adopt a single cutoff value to distinguish normal from elevated tHcy concentrations.

The measurement of plasma MMA is accepted as a sensitive functional marker of cobalamin deficiency/insufficiency and avoids the ambiguities associated with commonly used serum cobalamin assays [13]. As with tHcy, the upper limit above which an MMA concentration is considered to be elevated differs between laboratories [30] and a variety of approaches to determine reference intervals have been used. For example, Rasmussen et al. [31] reported an inner 95% reference intervals for MMA of 80–280 nmol/L established in 235 subjects prior to exclusion of those with a high probability of cobalamin deficiency (based on MMA decrease post cobalamin supplementation), outliers and those with high tHcy. In another study, an MMA concentration >470 nmol/L (inner 95%) was used as the upper limit in healthy adults with no clinical laboratory evidence of cobalamin deficiency [32].

Population-based reference intervals for 5-MTHF have not previously been reported. We are therefore unable to compare our findings with those of others. However the values for 5-MTHF concentrations obtained are in good agreement with data from other studies [6, 33]. In comparison with the median of 427 nmol/L (range: 92–1086) for red cell 5-MTHF from 109 healthy subjects, aged 18–65 years, reported by Smulders et al. [8], the values in our study were higher: median 560 nmol/L (range: 206–1110). This might be attributed to differences in methodologies and the fact that individuals with mild to moderate B-vitamin deficiencies were not excluded in that study [8].

A positive correlation between red cell 5-MTHF concentrations and age observed in this study is consistent with previous observations [34, 35]. Conversely, higher red cell 5-MTHF values in older participants are not reflected by a corresponding decrease in tHcy levels, suggesting that age is a folate-independent determinant of tHcy. It is not clear why folate concentrations are higher in the elderly. It has been suggested that this could represent an oversupply of the vitamin (diet) or it could reflect the tendency, as opposed to the younger group, to retain folate both in plasma and red blood cells [35]. Surprisingly, plasma 5-MTHF did not correlate with tHcy in our selected cohort used to establish 5-MTHF reference intervals. However, the whole blood 5-MTHF correlated weakly with tHcy (*P* = 0.043) and correlations of red cell 5-MTHF with tHcy were approaching statistical significance (*P* = 0.054). These weak or lack of correlations may be attributed to the relatively small sample size and the fact that all subjects with outlying tHcy and above our reference cutoffs were removed. To support this, Spearman's correlations (data not shown) on all subjects initially recruited *N* = 144 demonstrated stronger and expected significant correlations of plasma or red cell (whole blood) 5-MTHF with tHcy.

One caveat to our study is that the 5-MTHF reference intervals are unlikely to apply to children. Opladen et al. reported that serum 5-MTHF levels are the highest in the first year of life, followed by a continuous decrease until the age of 16 years [36].

## 5. Conclusions

In conclusion, the plasma and red cell 5-MTHF reference intervals for an adult population were determined from 126 subjects without evidence of functional folate and/or vitamin B_12_ deficiency as assessed by tHcy and MMA analyses. The application of these intervals will assist in the evaluation of folate status and facilitate the evaluation of 5-MTHF as an independent risk factor for disease states.

## Figures and Tables

**Table 1:** Summary of results of *N* = 126 subjects used to derive 5-MTHF reference intervals.

	Females *N* = 69 mean (SD); median	Males *N* = 57 mean (SD); median	Student's *t*-test *P* value	All *N* = 126 mean (SD); median
Age (range)	38 (10); 38 (23–60)	38 (12); 36 (19–64)	0.730	38 (11); 37
tHcy (*μ*mol/L)	8.4 (1.8); 7.9	9.7 (2.1); 9.7	<0.001	8.9 (2.1); 8.9
MMA (nmol/L)	116 (93); 101	112 (76); 100	0.794	114 (85); 100
Plasma 5-MTHF (nmol/L)	19.4 (8.5); 19.1	18.7 (8.2); 17.8	0.624	19.1 (8.3); 18.2
Redcell 5-MTHF (nmol/L)*	583 (222); 572	585 (164); 557	0.975	586 (197); 560
Whole blood 5-MTHF (nmol/L)	618 (229); 592	611 (172); 582	0.838	615 (205); 584

*Result adjusted for plasma 5-MTHF content.

**Table 2:** Reference intervals for 5-MTHF with 90% confidence intervals for lower and upper 95% reference limits.

Analyte	Reference intervals	Lower reference limit	Upper reference limit
Plasma 5-MTHF (nmol/L)	6.6–39.9	5.3–8.9	36.9–41.7
Redcell 5-MTHF (nmol/L)	223–1040	206–291	930–1110
Whole blood 5-MTHF (nmol/L)	245–1102	224–311	969–1184
